# GraFIX: A semiautomatic approach for parsing low- and high-quality eye-tracking data

**DOI:** 10.3758/s13428-014-0456-0

**Published:** 2014-03-27

**Authors:** Irati R. Saez de Urabain, Mark H. Johnson, Tim J. Smith

**Affiliations:** Centre for Brain and Cognitive Development, Birkbeck College, University of London, Malet Street, WC1E 7HX London, UK

**Keywords:** Fixation duration, Eyetracker methodology, Data quality, Infant, Naturalistic, Attention

## Abstract

Fixation durations (FD) have been used widely as a measurement of information processing and attention. However, issues like data quality can seriously influence the accuracy of the fixation detection methods and, thus, affect the validity of our results (Holmqvist, Nyström, & Mulvey, 2012). This is crucial when studying special populations such as infants, where common issues with testing (e.g., high degree of movement, unreliable eye detection, low spatial precision) result in highly variable data quality and render existing FD detection approaches highly time consuming (hand-coding) or imprecise (automatic detection). To address this problem, we present GraFIX, a novel semiautomatic method consisting of a two-step process in which eye-tracking data is initially parsed by using velocity-based algorithms whose input parameters are adapted by the user and then manipulated using the graphical interface, allowing accurate and rapid adjustments of the algorithms’ outcome. The present algorithms (1) smooth the raw data, (2) interpolate missing data points, and (3) apply a number of criteria to automatically evaluate and remove artifactual fixations. The input parameters (e.g., velocity threshold, interpolation latency) can be easily manually adapted to fit each participant. Furthermore, the present application includes visualization tools that facilitate the manual coding of fixations. We assessed this method by performing an intercoder reliability analysis in two groups of infants presenting low- and high-quality data and compared it with previous methods. Results revealed that our two-step approach with adaptable FD detection criteria gives rise to more reliable and stable measures in low- and high-quality data.

## Introduction

Every day of our life, we use our eyes to sample and create a perceptual image of the world around us. Without noticing, we manifest an array of eye movements that allow selecting and processing the parts of the visual field that are most relevant to us (Holmqvist et al., 2011). Fixations take place when the eyes remain relatively stable in a particular point, and it is only during these periods that visual encoding and processing occurs. Saccades, on the other hand, are the ballistic eye movements that take place between fixations when the eyes are rapidly moving from one point to the next. During these moments, visual sensitivity is suppressed (Matin, 1974).

Measuring and reporting fixation durations is common practice in experimental psychology (e.g., Henderson & Smith, 2009; Martinez-Conde, 2005; Martinez-Conde, Macknik, & Hubel, 2004; Nuthmann, Smith, Engbert, & Henderson, 2010; Tatler, Gilchrist, & Land, 2005). There is, in fact, a growing body of research that associates fixation durations with cognitive processes such as attention, information processing, memory, and anticipation (e.g., Castelhano & Henderson, 2008; Kowler, 2011; Malcolm & Henderson, 2010; Rayner, Smith, Malcolm, & Henderson, 2009; Richardson, Dale, & Spivey, 2007). While fixations may not be the only oculomotor event of interest to researchers measuring eye movements (e.g., researchers investigating attentional shifts may be interested in raw representations of saccadic or smooth pursuit trajectories), the majority of eye movement research assumes that gaze location can be equated to visual encoding of high-spatial-frequency foveal information, and for this to happen, the eyes need to be stable—that is, in a fixation (Rayner, 1998). The study of fixation durations is becoming increasingly important when investigating populations unable to follow the experimenter’s instructions, such as infants (e.g., Colombo & Cheatham, 2006; Frick, Colombo, & Saxon, 1999; Hunnius & Geuze, 2004; Hunter & Richards, 2011; Richards & Holley, 1999) or monkeys (e.g., Berg, Boehnke, Marino, Munoz, & Itti, 2009; Kano & Tomonaga, 2011a, 2011b). For instance, in a recent study, Papageorgiou et al. (2014) showed how individual differences in fixation durations in early infancy can predict individual differences in temperament and behavior in childhood, which can ultimately lead to early intervention practices that aim to improve executive attention and potentially identify infants at risk of attentional disorders such as ADHD.

Traditionally, researchers have used many different metrics to measure infants’ attention, such as familiarization or habituation procedures, preferential looking, or average looking times. These metrics, which should be applied to serve the experimental demands, are not always appropriate for answering certain questions, such as those concerned with the assessment of attention and information processing in spontaneous unconstrained settings (Aslin, 2007; Wass, Smith, & Johnson, 2013). In these cases, the analysis of fixation durations can help to gain valuable insights into the mechanisms underlying eye movement control.

Nevertheless, recent articles (Holmqvist, Nyström, & Mulvey, 2012; Wass et al., 2013) have highlighted the substantial impact that low-quality data can have on experimental measures. Poor eye-tracking recording can affect the validity of results, and sadly, it is still not common practice to report data quality measures or deeper descriptions of the fixation detection methods used (Holmqvist et al., 2011; 2012). This can alter the viability of research results and, hence, lead to problems replicating previous studies.

**Fig. 1:**
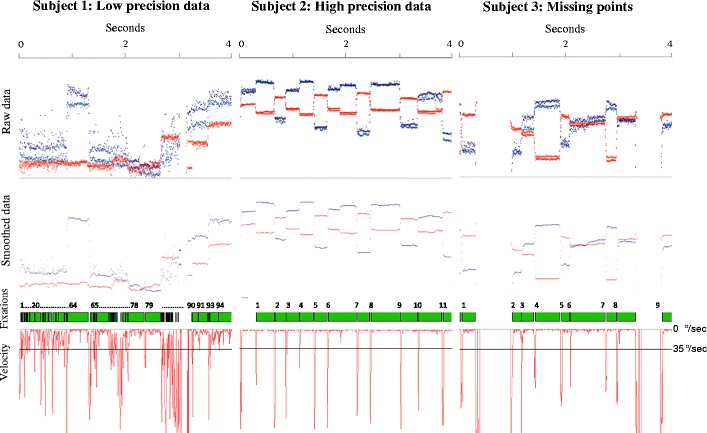
Data from 3 infant participants recorded with a Tobii TX300 at 120 Hz. The first and the second row show the raw and the smoothed data, respectively. The third row displays the fixations detected by a velocity-based algorithm (velocity threshold = 35°/sec), and the fourth the velocity calculated from the smoothed data. Participant 1 shows low-precision data, which is very common in young infants. As a consequence, the fixations-parsing algorithm detected a number of physiologically implausible artifactual fixations. Participant 2 displays high-precision data from infants. Although the algorithm was more accurate, due to the high velocity threshold, it merged together fixations that had short saccades in between (e.g., fixation 8). Participant 3 shows a participant that presents frequent missing data points

The raw data recovered from any eyetracker includes a time stamp and the *x*- and *y*-coordinates for one eye (monocular systems) or both eyes (binocular systems). Fixations can be identified when these coordinates are relatively stable in a point (and hence, the eyes’ velocity, defined as the rate of change in *x*- and *y*-coordinates from one gaze point to the next, is low), whereas saccades are flagged when the *x*- and *y*-coordinates are more variable in the scene and the eyes’ velocity exceeds a given threshold (see Fig. 1). Additionally, other types of eye movements can be detected in the raw data, such as smooth pursuit (Larsson, Nyström, & Stridh, 2013) or blinks (e.g., Morris, Blenkhorn, & Zaidi, 2002). In cases where the data quality and the sampling frequency are very high, it is even possible to identify very short fixational eye movements, such as microsaccades, glissades, or tremor (Nyström & Holmqvist, 2010).

## What is data quality and why it is so important

The quality of the raw data generated by the eyetracker may vary depending on many different factors, such as the eyetracker model and manufacturer, the eye physiology, the calibration procedure, the position of the participant relative to the eyetracker, the degree of head motion (Holmqvist et al., 2011, 2012), or even ethnicity (Blignaut & Wium, 2013). The term *data quality* entails different aspects affecting eye-tracking data, but not all these aspects will necessarily affect fixation detection equally.

Low data quality can have major effects on both spatial and temporal accuracy of gaze measurements. Spatial accuracy or offset refers to the difference in space between the detected gaze and the real gaze and can be an important issue when analyzing areas of interest (AOIs) (Holmqvist et al., 2012) . Apart from the vertical and horizontal accuracy that the eye-tracking systems report, aspects such as binocular disparity should also be taken into account, especially when studying special populations. For instance, we know that binocular disparity in young infants may be markedly larger than in adults (Appel & Campos, 1977; Yonas, Arterberry, & Granrud, 1987). However, it is also common to find infant data with very large disparities as a consequence of poor calibrations or incorrect angles between the eyetracker and the participant. Often, it is possible to minimize the effects of poor accuracy in AOIs analysis by simply enlarging the regions of interest or by quantifying and correcting the offset for each participant (Holmqvist et al., 2011, 2012). Frank, Vul, and Saxe (2012) designed an offline procedure to correct errors in calibration in order to increase the accuracy and include a measure that evaluates it. They presented their participants (infants from 3 to 30 months) with calibration points that appeared during the experiment and that were subsequently used to correct the offset.

Nonetheless, a data set with a large offset can also present high spatial precision and, thus, still be suitable for detecting fixations accurately. We refer to spatial precision as the consistency in detecting and calculating gaze points (see Fig. 1). Data sets with relatively low spatial precision will present higher gaze velocities as a result of noise in the data, and this will complicate the process of detecting fixations accurately. Precision can be affected by individual factors that vary on a participant basis (such as different eye physiologies or the position of the participant relative to the eye cameras), environmental factors that change according to the experimental design (such as the lighting conditions of the room where the participants are being tested), or the eye-tracking hardware and software (Holmqvist et al., 2011, 2012). The default spatial precision for a particular eyetracker can be calculated by using an artificial eye. Additionally, there are a number of methods for calculating spatial precision (Holmqvist et al., 2011, 2012), such as the root mean square (RMS) of intersample distances (commonly used by manufacturers) or the standard deviation, which measures the dispersion of each sample from a mean value. To see the effect that spatial precision has on the detection of fixations by a velocity-based algorithm, see Fig. 1.

Likewise, data loss (often a consequence of unreliable detections of the pupil or the cornea reflection) is another issue that can considerably affect fixation detection (Holmqvist et al., 2011, 2012; Wass et al., 2013). This also is highly dependent on individual and environmental factors, as well as on the eyetracker hardware and software. These individual variations in the recordings from different participants can lead to very different levels of data quality (accuracy, precision, and data loss), even when participants have performed the same study under the same experimental conditions. This can, in fact, be a problematic issue when trying to standardize the procedure to analyze the eye-tracking data: Do we use exactly the same protocol and values to analyze the data regardless of the noise that each participant presents? Or would it be more appropriate to adapt it somehow?

## Issues and recommendations when testing special populations: Infants

All these data quality problems can be particularly concerning if testing with special populations, such as infants or participants suffering from certain disorders. For instance, participants on the Autism spectrum may present a high degree of head movements (Kelly, Walker, & Norbury, 2013), and this can seriously affect the spatial precision and the accuracy of the data. Moreover, in some populations —such as in Parkinson patients—the head motion can be constant and consistent across all the participants for the study. Whereas some eye-tracking systems require the user to maintain the head still by using a chinrest (e.g., EyeLink), others allow a high degree of free head movements (e.g., Tobii or SMI eyetrackers). However, these systems include some extra algorithms for the head position calculations that can interfere with the gaze estimation and, hence, affect spatial precision (Holmqvist et al., 2011; Kolakowski & Pelz, 2006).

Infants constitute a group that can be especially challenging to test in eye-tracking studies. As well as presenting a high degree of head movement, especially from 7 to 8 months of age, when their locomotor abilities are rapidly improving (Adolph & Berger, 2006), many of the quality problems are derived from poor calibration procedures. Traditionally, infants are calibrated using five-point calibrations where a colorful puppet is presented in each corner and center of the screen, while adults usually perform a nine-point calibration following a series of small dots.

The first obvious problem occurs when the infant does not look at the calibration points when they are presented. As a consequence, the offset calculation will be erroneous, and the spatial accuracy will be affected in one or more areas of the screen. Sometimes, infants do not consistently look at all the points, because they are simply not interested in them (e.g., after performing several calibrations in a row, when the infant is already familiarized with the stimuli). To minimize these issues, the researcher can regularly change the calibration stimuli (e.g., varying it in colors and/or shapes) and accompany the points’ presentation with attractive sounds. At other times, the calibration points are presented too briefly for the infant to move his/her eyes from one point to the next. This problem is more obvious in participants younger than 4 months, since the neural structures implicated in oculomotor control are still underdeveloped (Johnson, 2010; Johnson, Posner, & Rothbart, 1991). This is manifested through long disengagement latencies, also known as *sticky fixations* or *obligatory attention* (e.g., Hood & Atkinson, 1993). This tendency lessens as the infant develops, and by around 4 months of age, they are able to shift their attention more rapidly and accurately from one point to another. However, during the first months of life, if the presentation of the calibration points is relatively quick, the infant may not be able to follow them. This problem can be solved by giving the researcher the possibility to decide when the next calibration point should appear; for example, the researcher presses a button when the infant is looking to the current calibration point.

Visual acuity does not reach adultlike levels before the third year of life (Atkinson, 2000). In fact, newborns are believed to have a fixed depth of focus (Salapatek, Bechtold, & Bushnell, 1976), and in many cases, they even present some degree of astigmatism (Atkinson, 2000). However, it is during the first 3–4 months that the biggest changes in visual acuity, contrast sensitivity, and focusing ability (accommodation) occur. This means that during the first months of life, infants may have problems accommodating as a function of target distance, and hence, they may not see objects (i.e., the calibration points) that are farther away than a certain distance (Salapatek et al., 1976). Thus, for participants younger than 3–4 months, the distance between the infant and the presentation screen needs to be considerably shorter (around 30–40 cm; Salapatek et al., 1976) than the distance that is recommended for most eye-tracking systems (around 60 cm; Holmqvist et al., 2011). A change of the recommended distance will obviously affect the quality of the data.

Another problem related to the calibration procedure is the size of the calibration points. As was previously explained, infant calibration targets are usually bigger and visually more complex than the traditional adult targets. This means that even though it is possible to tell whether the infant is foveating the correct object, we still cannot know which part of the object. Once again, this can lead to imprecise offset calculations. A way to minimize this problem is to use calibration points that, even though they are bigger than the typical dots for adults, have a clear central point that is more likely to be gazed (e.g., a colorful spiral) or decreases in size down to a point.

A final problem related to the calibration procedure is the viewing angle between the eyetracker and the participant. Wrong angles may produce higher offsets and inaccurate binocular disparities. Usually, this problem can be solved repeating the calibration procedure once again, changing the participant’s or the eyetracker’s position each time. Nevertheless, infants do not respond to instructions, and their attention span is considerably lower than adults’. Thus, repeating this procedure may not always be feasible.

Most of the current eyetracking systems use corneal reflection to estimate gaze, where the pupil is detected using bright or dark pupil techniques. In bright pupil techniques, the pupil looks bright as a consequence of on-camera-axis illumination, whereas in dark pupil techniques, the pupil looks dark due to off-camera-axis illumination (for a wider review, see Holmqvist et al., 2011). The illumination of an IR light creates a bright glint on the back of the cornea that can be detected by computer vision algorithms. It is this glint and its distance relative to the center of the pupil that is used to estimate the gaze on the screen. There are various reasons why the glint or the pupil can be unreliably detected or not detected at all (e.g., poor lighting conditions, different eye physiologies, wrong distance and/or angle between the participant and the eyetracker). As a consequence, the data quality can be seriously affected (poor spatial precision, missing points), and hence, it can complicate the process of identifying fixations (Holmqvist et al., 2012).

Infants’ eyelids can be particularly watery, especially during the first few months of life, and this can considerably interfere with the glint detection process. Usually, bright pupil techniques are considered to be more accurate than dark pupil ones when dealing with certain eye physiologies, like bright eyes or watery eyelids (Gredebäck, Johnson, & von Hofsten, 2009). For this reason, when testing young infants, it is always a better choice to use eye-tracking systems that also include a bright pupil corneal reflection technique.

## Previous methods for detecting fixations

Fixations can be detected by an algorithm or by a person on the basis of visual inspection of the raw eye-tracking data. Many eyetracker manufacturers already provide smoothing and event detection tools. However, what these algorithms do to the data may still be unclear for many users, especially for those not particularly familiar with event detection techniques. Additionally, when the user chooses arbitrary input parameters without considering issues like data quality, the sampling frequency, or other aspects of the experimental design (e.g., it is not the same to detect small fixational eye movements in reading research or to detect saccades in infants), the detection results can be gravely affected, and hence, the validity of the experimental outcomes can be questioned (Holmqvist et al., 2012).

Event detection algorithms can be classified into two main groups: dispersion and duration algorithms and velocity and acceleration algorithms (for more detailed reviews, see Holmqvist et al., 2011). Dispersal-based algorithms use a minimum fixation duration threshold (e.g., 50 ms) and the positional information (dispersion) of the eye-tracking data in order to decide whether consecutive points belong to the same fixation—in which case, they are grouped together. If not, they are assumed to be a saccade or a missing point. Dispersion can be measured according to different metrics (Blignaut, 2009), such as the distance between the points in the fixation that are the farthest apart (Salvucci & Goldberg, 2000), the distance between two random points in a fixation (e.g., Shic, Scassellati, & Chawarska, 2008), the distance between two points at the center of a fixation (e.g., Shic et al., 2008), the standard deviation of *x*- and *y*-coordinates (e.g., Anliker, 1976), or a minimum spanning tree of the points in a fixation (e.g., Salvucci & Goldberg, 2000). Currently, it is possible to find a number of commercial (e.g., SMI BeGaze) and noncommercial implementations for these algorithms (e.g., Salvucci & Goldberg, 2000), which are mostly used to parse low-sampling-rate data (< 200 Hz). On the other hand, the algorithms from the second group calculate the velocity and/or acceleration for each point in order to detect events on the data. Velocity-based algorithms, in particular, flag all the points whose velocity are over a threshold (e.g., 10–70 °/sec) as saccades and define the time between two saccades as a fixation. Once again, there are a number of commercial (e.g., Tobii, EyeLink) and noncommercial (Nyström & Holmqvist, 2010; Smeets & Hooge, 2003; Stampe, 1993; Wass et al., 2013) variations for this type of event detection algorithms. These algorithms are commonly used in data collected at high sampling rates (e.g., >500). All these algorithms are very sensitive to noise, and unless the collected data have a very high spatial precision, the results will include a number of artifactual fixations.

The use of event detection algorithms implies decisions about which thresholds should be selected in order to obtain optimal results. However, how these decisions are made, the range of parameters that can be manipulated, and whether they are reported in published papers is not yet standardized, making it difficult to compare or replicate results from different studies. Komogortsev, Gobert, Jayarathna, and Gowda (2010) compared the performance of different velocity- and dispersal-based algorithms and presented a standardized scoring system for selecting a reasonable threshold value (velocity or dispersion threshold) for different algorithms. Nevertheless, this article did not take into account the individual differences in data quality across participants and/or trials.

Most researchers tend to use the same input parameters for all the participants, paying very little attention to these variations in data quality and the effects that selecting different thresholds may have on the data from different participants. Nyström and Holmqvist (2010) presented a new velocity-based algorithm for detecting fixations, saccades, and glissades, using an adaptive, data-driven peak saccade detection threshold that selects the smallest velocity threshold that the noise level in data allows. The use of thresholds was motivated by physiological limitations of eye movements. These algorithms already highlighted the importance of adapting the input parameters to different levels of noise but still did not solve the problem of accurately detecting fixations in data sets with relatively higher levels or noise, such as those from infants.

Wass et al. (2013) analyzed standard dispersal-based fixation detection algorithms and showed how results were highly influenced by interindividual variations in data quality. Additionally, they went a step further to solve these problems, developing new detection algorithms that include a number of post hoc validation criteria to identify and eliminate fixations that may be artifactual. These algorithms already exclude many artifactual fixations that were included when other velocity-based detection algorithms were used. However, any automatic approach for detecting fixations in data with a certain degree of noise are likely to produce artifactual fixations that are erroneously calculated and/or fixations that are not detected at all.

An alternative to using automatic algorithms is to hand-code eye movements on the basis of a visual inspection of the data. For instance, developmental psychologists have traditionally studied infants’ attention and eye movements by video-taping participants and hand-coding the direction of the gaze post hoc (e.g., Elsabbagh et al., 2009). Also, it is a common practice, when analyzing the data from head-mounted eyetrackers, to replay the scene and eye videos frame by frame and make annotations of the onsets and offsets of fixations on a separate file (e.g., Tatler et al., 2005). Obviously these techniques are highly time consuming and can limit the number of participants that a researcher is able to test and code.

With a view to avoiding these problems, some researchers have suggested excluding all participants whose spatial precision is over a predefined threshold (Holmqvist et al., 2011, 2012). This way the use of automatic algorithms should be relatively safe, although not perfect. However, excluding participants according to their data quality is a luxury that not every study can afford. As was previously explained, the data quality for many experiments studying high-cost populations such as infants or special populations to whom access is limited may be consistently low. Using data quality as an inclusion criterion might result in many participants (or even all) being excluded. In cases of special populations, the data can be too valuable to be discarded.

To address these issues, we developed GraFIX, a method and software that implements a two-step approach: Fixations are initially parsed by using an adaptive velocity algorithm, then hand-moderated using a graphical easy-to-use interface. This method aims to be as fast and accurate as possible, giving the researcher the possibility of fixing and adapting the algorithm’s outcome in order to remove all the artifactual fixations manually and include those that were not accurately detected. The automatic detection algorithms include a number of post hoc validation criteria aiming to obtain the cleanest results that the algorithms alone permit, in order to facilitate and speed the process of hand-coding fixations.

## Introducing GraFIX

GraFIX is a multiplatform application developed in C++ and QT frameworks that makes use of Armadillo C++ linear algebra library. It works with any binocular or monocular eye-tracking system that can record raw X/Y gaze coordinates, including SMI, EyeLink or Tobii eyetrackers.

The present application implements a two-step approach where fixations are initially parsed by using an adaptive velocity-based algorithm, before the algorithm’s outcome is hand-moderated using a graphical interface. Previous methods for detecting fixations have adopted either a purely automatic approach or manual coding. Due to the high variability in data quality across participants and even within a single participant (e.g., as a result of moving the head throughout the eye-tracking session), the automatic detection algorithms can be remarkably unreliable. On the other hand, current hand-coding methods (e.g., coding fixations looking at the videos frame by frame) can be extremely time consuming and, in some cases, imprecise if coding low-quality data sets.

The proposed method combines these two approaches together in order to detect fixations in a rapid manner and obtain a fixation distribution with the lowest possible degree of noise. The present fixation detection algorithm includes a number of input parameters that can be easily adapted on a participant basis. Additionally, it implements three post hoc validation criteria that fix or remove many of the artifactual fixations generated by the velocity-based algorithm. The ultimate aim of adapting the input parameters and applying certain post hoc validation criteria is to obtain the most accurate outcome by the algorithms alone and, thus, reduce the hand-coding time during the subsequent step. Once the fixations have been automatically estimated, the researcher can evaluate them and fix those that were not accurately detected using the GraFIX graphical hand-coding tool.

**Fig. 2:**
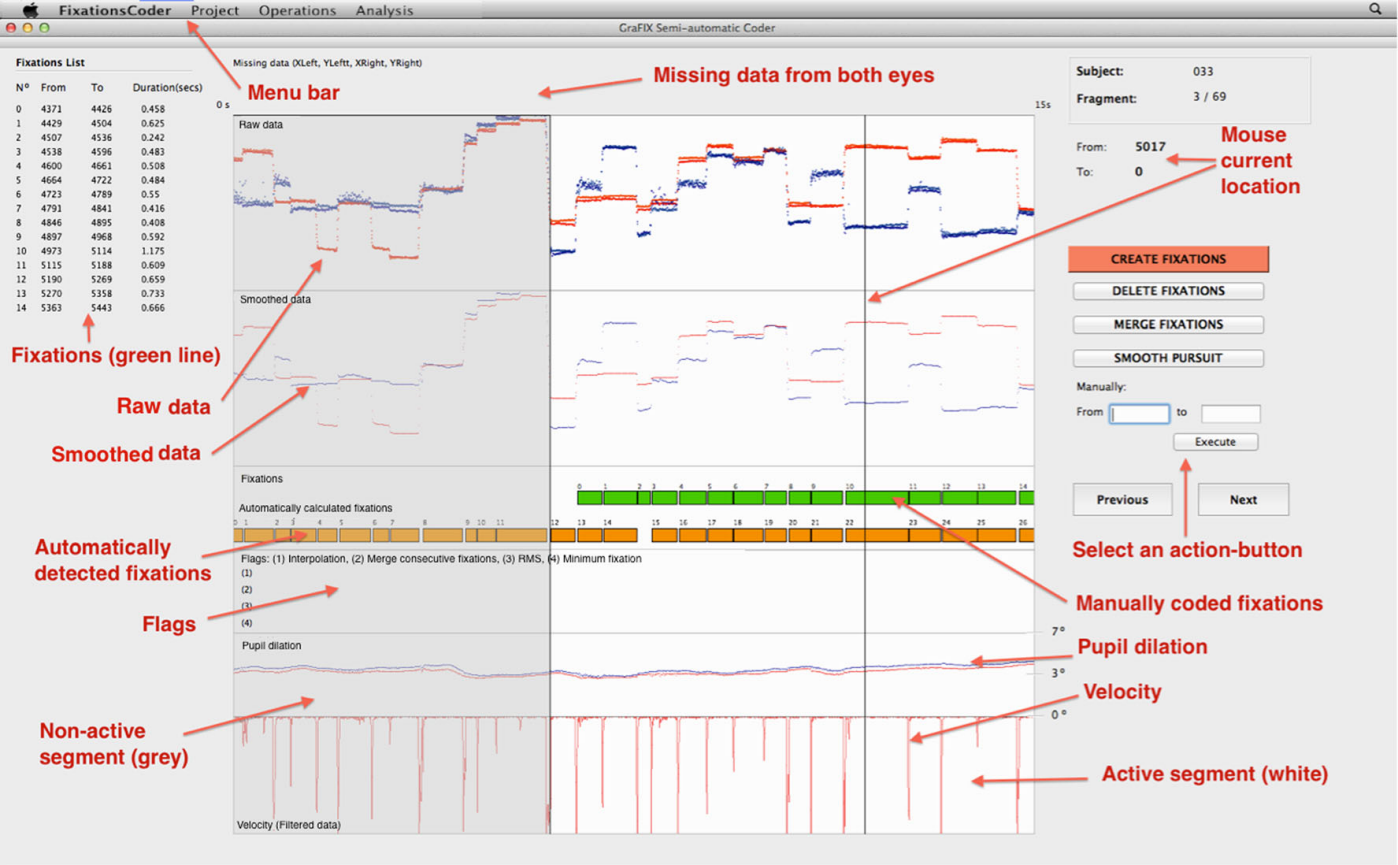
GraFIX application overview. This is the main window where the user is allowed to manipulate fixations by looking at the eye-tracking data, which are displayed in their different formats (raw and smoothed data). Top section of window: *x*- and *y*-coordinates are presented on the vertical axis, and time on the horizontal axis. Fixations can then be identified when both *x*- and *y*-coordinates do not present any displacement in the vertical axis, and saccades when there is a vertical displacement between two fixations accompanied by a velocity peak. Automatically detected fixations (orange rectangles) are displayed aligned with hand-moderated fixations (green rectangles), which are the ones that can be manipulated by selecting an action on the right side of the screen (create, delete, or merge fixations; code them as smooth pursuit) and mouse-clicking on them. Furthermore, GraFIX allows defining and indicating the sections where the user is interested in detecting fixations by displaying them on white or gray

GraFIX displays the eye-tracking coordinates in the raw and the smoothed data boxes (see Fig. 2; for an extended explanation of the user interface, see Appendix 1). It presents the *x*- and *y*-coordinates on the vertical axis, and time on the horizontal axis. Fixations can then be identified when both *x*- and *y*-coordinates do not present any displacement in the vertical axis, and saccades when there is a vertical displacement between two fixations accompanied by a velocity peak (see Fig. 2, velocity box). Occasionally, our eyes move to smoothly pursue an object in the visual scene, and this type of eye movement can be identified when there is a regular increasing or decreasing displacement in the vertical axis with low velocity and acceleration (not present in Fig. 2).

The following sections will present a detailed review for the present two-step approach for fixation detection.

## Automatic detection of fixations

**Fig. 3:**
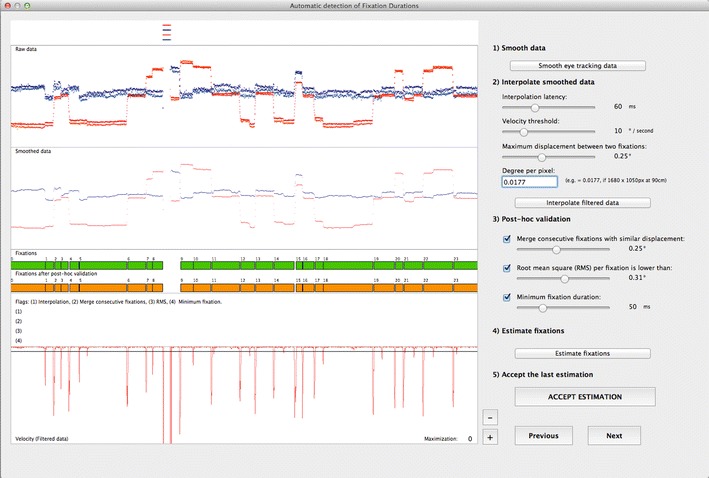
GraFIX Automatic detection of fixations. This screen displays the input parameters for the automatic detection. It is possible to adapt the parameters by simply changing their values from the sliders. When *Estimate fixations* is pressed, GraFIX executes the detection algorithms and displays the results on the orange rectangles. Flags indicating which post hoc validation criterion was executed are also displayed. This process is relatively fast and, thus, allows multiple and easy adjustments of the parameters. Once the user is satisfied with the results, the detection can be accepted by pressing *Accept estimation*. This will copy the automatically detected fixations (orange) on the hand-modulated fixations area (green)

The first action for the two-step approach to detecting fixations is to parse the eye-tracking data using adaptive velocity-based algorithms. The present automatic detection algorithms (1) smooth the raw data, (2) interpolate missing data points, (3) calculate fixations using a velocity-based algorithm, and (4) apply a number of post hoc validation criteria to evaluate and remove artifactual fixations (to see the pseudo-code, go to Appendix 2). The input parameters (e.g., velocity threshold, interpolation latency) can easily be manually adapted to fit the data from different participants that present different levels of data quality (see Fig. 3).

The objective of these algorithms is to obtain the most accurate fixation detection for each participant and, thus, reduce the amount of time spent manually correcting fixations in the subsequent step.

### Smoothing the data

GraFIX uses a bilateral filtering algorithm in order to decrease the noise levels from the raw data. The present version of the algorithm is based on previous implementations (Durand & Dorsey, 2002; Frank, Vul, & Johnson, 2009) that average the data for both eyes and eliminate the jitter, while preserving saccades.

If only one of the eyes is detected, GraFIX allows the user to decide whether the detected eye will still be smoothed or the sample should be excluded. Previous researchers have argued that when one eye is not detected, the data from the other eye may be unreliable (Wass et al., 2013). However, when the eye-tracking data comes from special populations, such as infants, the fact that one of the eyes is not detected does not necessarily mean that the sample from the other eye is inaccurate. For instance, it can be the case that the infant is simply occluding one of his/her eyes with his/her hand, causing difficulties for the accurate detection of both eyes. Occasionally, these missing points could lead to inaccurate results regardless of the inclusion or exclusion of the data (e.g., if one eye was occluded during a fixation).

Unless the data come from adult populations, we recommend including the samples in which only one eye is available —in the interest of obtaining the highest number of fixations. Once the fixations are automatically calculated, it is possible to manually remove the artifactual fixations (such as those generated as a consequence of occluding one eye during a fixation).

### Interpolating smoothed data

Occasionally, a data set will present a number of short gaps of missing data where the eyes are not accurately detected. These gaps can range from 1 to even 150 ms and may severely affect the detection of fixations. To address this problem, we include an algorithm that interpolates short segments of missing data.

Clearly, we do not want to interpolate every single segment of missing data: The algorithm will only fill the gaps that are shorter than a given threshold and that belong to a fixation, and not a saccade. The *interpolation latency* is the longest period of missing data that will be interpolated. This value may change depending on our data quality or the experimental design. For instance, for our infant studies, we use an interpolation duration of 60 ms, since the shortest fixations that could be manually coded in our data were never longer than this value. Nevertheless, other researchers have also used interpolation latencies as long as 150 ms, arguing that the minimum time taken to program a saccade is 100–130 ms and, hence, this way it is possible to avoid interpolating through a complete saccade–fixation–saccade sequence (Wass et al., 2013).

**Fig. 4:**
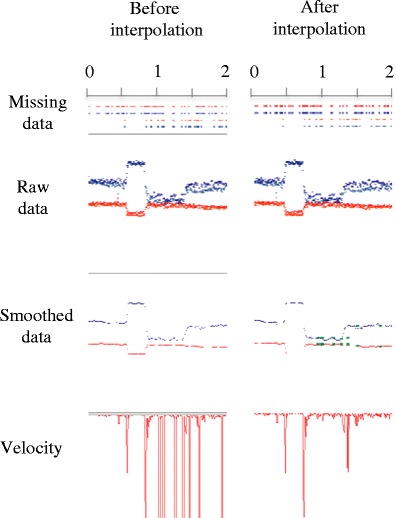
Interpolation. The sample on the left shows a fixation that has missing points. As a consequence, there are velocity peaks in the middle of the fixation. On the other hand, the picture on the right shows the same sample after interpolating the missing points (the green dots on the smoothed data are the interpolated points). In this case, the velocity calculation looks significantly cleaner

First, the interpolation algorithm flags all the samples in the data whose velocities lie over the velocity threshold as saccades, and the data segments between two saccades are targeted as fixations. The *velocity threshold*, which is also used in the forthcoming steps, can be set to meet the requirements of different data sets (see Fig. 4).

Second, when a gap longer than the interpolation latency is found, the algorithm finds the subsequent and previous fixations and calculates the mean Euclidean distances from a central point for each of them. When the difference between both Euclidean distances is smaller than the *maximum displacement between the two ends of a fixation*, the gap is interpolated. It is important to determine the correct *degree per pixel* parameter (in visual angle) in order to convert the degrees to pixels properly.

### Velocity threshold and fixation detection

As in previous velocity-based detection algorithms (e.g., Nyström & Holmqvist, 2010; Smeets & Hooge, 2003; Wass et al., 2013), all the samples whose velocities lie over a certain threshold are flagged as saccades and the data segments between two saccades are targeted as fixations. Choosing the right velocity threshold highly depends on the characteristics of the data that are being analyzed or on how short the saccades that need to be detected are (Holmqvist et al., 2011). For instance, low sampling rates will present some limitations when detecting very fast eye movements, such as microsaccades. Previous research has shown that saccades smaller than 10° cannot be detected with systems with a sampling rate of 60 Hz and lower (Enright, 1998). This is because the peak velocity calculation may not be accurate enough if only a very few samples of a saccade were recorded. The lower the sampling frequency, the lower the calculated velocities for short saccades will be. In these cases, a fixation detection algorithm would merge the fixations before and after an undetected saccade, and this would result in longer artifactual fixations. In order to reliably detect small saccades and reduce noise, it is recommended to use high sampling frequencies and lower velocity thresholds (see Fig. 4).

Nevertheless, data from special populations such as infants can still represent a challenge, due to their low quality. Thus, when the noise levels are higher, the velocity threshold should be increased accordingly in order to decrease the number of “false positive” saccades. This leads to the question of whether different noise levels in a given data set could entail the inaccurate detection of fixations in low- or high-quality data making it difficult to compare and group participants together. As has been suggested in previous research (Wass et al., 2013), different levels of noise can seriously affect the outcome from fixation detection algorithms. There are two opposing approaches that have been traditionally used to minimize this issue. Some researchers prefer to use exactly the same input parameters for all the participants (such as the velocity threshold), regardless of the level of noise each participant presents (Wass et al., 2013). Usually, these parameters are set to fit the requirements for participants with high levels of noise. Consequently, the velocity threshold can be too high to detect relatively fast saccades, which could ultimately lead to the detection of long artifactual fixations. Moreover, these saccades would still remain undetected in very low-precision data sets even after lowering the velocity threshold. On the other hand, it is possible to adapt the input parameters according to the level of noise on a participant-by-participant basis (e.g., Nyström & Holmqvist, 2010). Although the use of different velocity thresholds can lead to different outcomes from the detection algorithms, it is possible to remove a number of artifactual fixations (this is especially important in noisy data sets) by also adapting the input parameters for the post hoc validation criteria (explained below) and manually fixing the fixations that were not detected correctly. With current systems, it will never be possible to remove all the noise, but we can minimize its effects and obtain the cleanest result for both low- and high-quality data.

### Post hoc validation

Once the data are smoothed and interpolated, fixations can be automatically calculated by executing the velocity-based algorithms described below. Additionally, it is possible to apply a number of post hoc validation criteria in order to manipulate the algorithm’s outcome and obtain the most accurate results. All the input parameters (including the velocity threshold described in the previous section) can be adapted on a participant-by-participant basis. This way, it is possible to personalize the detection process to different participants with different levels of data quality and reduce, to a certain degree, the insertion of noise by the algorithms.

#### Merging adjacent fixations with similar location

**Fig. 5:**
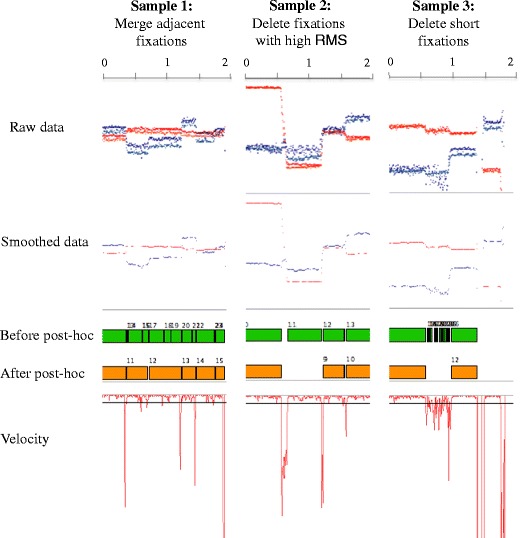
Post hoc validation examples. We present different segments of data from one infant, collected with a Tobii TX300 system at 120 Hz. The velocity threshold was set to 9 °/sec. Sample 1 shows how, as a consequence of the low precision in the data, there are velocity peaks that pass the velocity threshold and, hence, are erroneously flagged as saccades. Merging adjacent fixations with similar locations helps overcome this problem. Sample 2 shows fixations with low precision that are deleted if they overcome a given RMS threshold. Sample 3 shows a number of very short artifactual fixations that are detected as a consequence of the poor precision in the data. The minimum fixation post hoc validation criterion deletes all the fixations shorter than a given threshold

Occasionally, the detection algorithms can break down fixations with low precision into a number of smaller fixations. This is because one or more samples from a fixation may present a velocity peak that is higher than the defined velocity threshold and, hence, be mistakenly flagged as a saccade (see Fig. 5, first column). As such, even fixations with high spatial precision can have few samples that were not accurately recorded and, consequently, will generate the same peaks in velocity as saccades. Therefore, this issue is of special concern in low-spatial-precision data sets, even though it can affect any eye-tracking recording.

The present post hoc validation algorithm merges adjacent fixations that are close in time and space (see Fig. 5, first column). First, the algorithm will select fixations that have a gap in between of less than 50 ms, and then it will evaluate whether these fixations are also close enough in space. For this purpose, the user is able to set up a threshold and define the maximum distance in degrees between two fixations. Thresholds for merging should depend on the sampling frequency, data quality, stimulus spacing, density of visual information, or even on the research question (e.g., studies investigating microsaccades may require very low thresholds). Participants that present low spatial precision, for instance, will require higher distance thresholds than those with high-precision samples. However, we do not recommend using thresholds over 0.45°, since the algorithm may merge together fixations that have short saccades in between. In segments where the spatial precision is particularly low, we recommend excluding the detected fixations, rather than increasing this threshold. In high-precision data recorded with a high sampling rate, such small displacements between fixations may actually represent microsaccades, and therefore, post hoc merging of fixations should be avoided.

#### The root mean square of intersample distances per fixations does not exceed a threshold

Spatial precision is crucial for a correct detection of fixations (Holmqvist et al., 2011, 2012)—in particular, if the research interests rely on the study of very small fixational eye movements, such as microsaccades. Furthermore, as was explained in the sections above, in populations such as infants, spatial precision can also be a major issue, as a result of the particularities of their eyes and the difficulties that this can entail when using corneal reflection techniques.

The present post hoc validation criteria calculate the RMS for each fixation for both the vertical and horizontal axes together and delete all the fixations with a value above a given threshold (in degrees of visual angle) (see Fig. 5, second column). In high-precision data sets, the RMS can be smaller than 0.10°, while in low-precision data sets, it can be higher than 0.20°.

#### Minimum fixation duration

Especially in data sets with low spatial precision, the detection algorithms may mistakenly generate very short fixations. This is because the dispersion for some of the samples belonging to a fixation may be high enough to generate velocity peaks over the given threshold that will be flagged as saccades (see Fig. 5, third column).

In order to avoid this problem, GraFIX can delete all the fixations with a duration under a given threshold (e.g., 100 ms). In the event that a data set is very noisy, the minimum fixation threshold should be set higher. This does not necessarily mean that it is not possible to find short fixations in our data set: In case there are clean short fixations, they can also be manually coded after the automatic detection of fixations.

### Defining the right parameters for detecting fixations

GraFIX is able to run the fixation detection algorithm and visualize the results very quickly. For instance, 30 min of data at a sampling frequency of 300 Hz can be parsed in less than a second (the processing speed also depends on the capabilities of the machine where the application is being executed). This permits the user to adjust the input parameters and evaluate their effect on the data in a rapid manner. In fact, it can be immensely helpful (for novice users in particular) to be able to visualize each estimation and see how changing the input values described above will affect the detection of fixations.

When the user acknowledges that the fixation detection is accurate enough, the estimation can be accepted, and the manual inspection of the algorithms’ outcomes will start. At this point, the user can review all fixations and manipulate them in order to meet a chosen fixation detection criterion and reduce the noise in the data.

The researcher can decide whether the input parameters for the event detection algorithms should remain the same for the entire data set or whether they should change as a function of data quality. In case different parameters are used for different participants at different levels of data quality, it can be argued that since the results for these participants were calculated by using different criteria, they should not be grouped together. We know, however, that the selection of certain input parameters will affect low- and high-quality data differently (see the Velocity Threshold and Fixation Detection section). Therefore, even when the parameters remain the same for the entire data set, they may affect the results from participants presenting high and low data quality differently. For this reason, it can also be argued that adapting the input parameters is a step in reducing the levels of noise for each participant’s results. Nonetheless, the algorithms alone, even after adapting the input parameters on a participant basis, are always subject to errors, particularly when processing low-quality data. In order to fix some of these errors and achieve the cleanest results possible, we also propose the manual adjustment of fixations.

## Manual adjustments of fixations

Once the fixations have been automatically calculated, the user can examine and manipulate them in order to fix the algorithms’ outcome. Even the most accurate algorithms may generate a number of artifactual fixations that can corrupt the validity of the experimental results. This is because most of the time, the data are assumed to have high spatial precision or, at least, to present similar levels of noise across the whole duration of the experiment, and this is not always the case. In fact, when working with populations such as infants, it will rarely be the case.

Fixations can be created, deleted, or merged by simply clicking and dragging the mouse on the main screen. For instance, in order to create a fixation, the user just needs to click the point on the screen where the fixation starts and drag the cursor until the point where the fixation ends. The tags *From* and *To*, located at the upper right of the screen, indicate the exact onset and offset of the current fixation (see Fig. 2). Once the fixation is created, it will appear on the fixations list at the upper left of the screen. If fixation A starts on the current fragment and ends on the next, we need to (1) create a first fixation whose onset fits with fixation A’s onset and drag the cursor a bit further than the end of the fixations box, (2) create a second fixation on the next fragment whose offset fits with fixation A’s offset, and (3) merge both fixations. Additionally, fixations can be coded as smooth pursuit once they are created.

In general, high-quality data sets will not need as much manual adjustment, whereas low-quality sets will require significantly more. We refer to the process of first parse fixations applying detection algorithms and then fix the outcome with the hand-coding tool as the two-step approach. The only difference between this method and hand-coding is that for the two-step approach, the detection algorithms are first executed and their output is used as a starting point for doing the manual coding. Thus, the results from the two-step approach and from a purely hand-coding approach should be approximately the same, while the coding-time will be considerably reduced with the proposed method (for more details, go to the *Software validation*, *Comparing hand*-*coding with the two*-*step approach* section). To demonstrate the time difference, we coded a randomly selected participant both manually (using GraFIX hand-coding tools) and by applying the two-step approach. The total length of the experiment was 18.5 min. The coder invested considerably more time hand-coding the data (51 min), as compared with applying the two-step approach (35 min). Still, these values are both considerably lower than the time that was required by previous hand-coding approaches (e.g., coding the same videos frame by frame could easily take several hours).

Evidently, the amount of time that the researcher needs to expend coding depends on his/her expertise and on the characteristics of the data (e.g., data quality). Furthermore, accurate detections will require less coding than inaccurate ones, and hence, the coding time in these cases will be shorter.

## Visualizations

Most of the time, it is relatively easy to identify fixations by looking at the 2-D representation of the *x*- and *y*-coordinates; however, when the coder is not entirely sure about coding a particular fixation, it is very helpful to visualize the data in other formats.

GraFIX allows the 2-D visualization in real time of the raw and smoothed data together with the IDs of the fixations that are being coded. Additionally, it is possible to include the stimuli in the background for all the different tasks of the experiment. This permits a further evaluation of the fixations and facilitates the coding process, especially for novice coders.

## Pupil dilation

GraFIX will also process the pupil dilation data, in case they are provided. Once the pupil dilation data are included in the raw input file, they are automatically displayed on the main window. Furthermore, the visualization dialogs include the option to play the eye-tracking data together with pupil dilation. Each fixation that is created or modified by GraFIX includes the pupil dilation means.

## Software validation

GraFIX has been evaluated from four different perspectives. First, the agreement between two different raters was assessed using the intraclass correlation coefficient (ICC) in two groups of infants featuring low- and high-quality data. Second, hand-coding results were compared with those of the two-step approach (automatic detection + hand-coding), demonstrating that both techniques were generating exactly the same results. Third, the outcome from GraFIX automatic algorithms was compared with that of the two-step approach. Finally, we compared hand-coding results with GraFIX automatic algorithms and previous automatic detection algorithms (the velocity-based algorithms from Wass et al., 2013; the adaptive velocity-based algorithms from Nyström & Holmqvist, 2010; and the I-VT filter velocity-based algorithm as implemented in Tobii-studio 3.0.0).

Additionally, GraFIX has been successfully used to code data from various monocular and binocular eye-tracking systems, such as Tobii, Eye Link, or SMI systems, at different sampling rates. Even though using different eyetrackers does not affect the performance, high sampling rates may slow down the execution of the algorithms.

### Intercoder reliability

Manual coding always involves an evaluation of the degree of agreement between different raters. Data from a group of three infants with low spatial precision (RMS > 0.30° per infant; 20–25 min of data each) and another group of three infants with high spatial precision data (RMS < 0.13° per infant; 20–25 min of data each) were recorded using a Tobii TX300 eyetracker at a sampling rate of 120 Hz and MATLAB (with Psychophysics Toolbox^1^ Version 2 and T2T^2^). Note that even though the spatial precision for the second group was relatively high, it was still data coming from infants, and thus, there was a high degree of head motion and frequent missing data points.

An external coder with no eye-tracking experience and naive as to expected outcomes was trained to code fixations from both groups. The second coder was one of the authors of this article. The coders had to (1) run the automatic detection algorithms, using the parameters from Table 1, and then (2) manipulate the resulting outcome in order to remove artifactual fixations or add those undetected by following the predefined guidelines. The input values for automatic detection were chosen after executing the algorithms with a wide range of values and evaluating the outcomes. The values from Table 1 may not necessarily be optimal in data sets with other characteristics and may change for different participants, experiments, and/or groups. We decided to have two sets of parameters for the two different groups in order to facilitate the process for the novice coder and to define some standards for the execution of the automatic detection algorithms.

**Table 1:** Input parameters for high- and low-spatial-precision data for the intercoder reliability data

	High Spatial Precision	Low Spatial Precision
Interpolation latency (ms)	60	60
Velocity threshold (°/sec)	9	20
Maximum interpolation displacement (°)	0.25	0.25
Degree per pixel (°/pix)	0.0177	0.0177
Maximum distance for merging adjacent fixations (°)	0.24	0.35
Maximum time for merging adjacent fixations (ms)	50	50
Maximum RMS per fixation (°)	0.24	0.21
Minimum fixation duration (ms)	99	120

In order to keep the same standards across participants and coders, it is essential to define strict guidelines about how to code the data. A fixation was coded when both the *x*- and *y*-coordinates were stable at one point, or in other words, when the 2-D representation of both *x*- and *y*-coordinates were displaying horizontal lines. If the detection of one eye was imprecise, the data from the other eye were used. If the coder was not entirely sure about coding a particular fixation, he or she was advised to leave it out. Saccades that were too short to be detected by the algorithms were also coded. Fixations that were cut by blinks and smooth pursuit eye movements (diagonal movement of the X/Y trace) were deleted. These guidelines may change depending on the experimental design. For instance, if the researcher is particularly interested in smooth pursuit eye movements, those would not be deleted.

The interrater reliability between the means and the number of detected fixations was evaluated using the ICC. A strong agreement between the mean fixation durations was found for both the low-quality data group (with an ICC of .967, *p* = .016) and the high-quality data group (with an ICC of .887, *p* = .038). Additionally, we also found strong agreements in the number of fixations detected for low-quality (with an ICC of .938, *p* = .037) and high-quality (with an ICC of .971, *p* = .009) data. Interestingly, the agreement in the low-quality group is slightly higher than in the high-quality group. This may be because fixations that were not clear enough were not coded, and this can appear to be slightly more subjective in high-quality data sets, where the data quality is a bit more variable across the time course of the experiment (due to head motion and/or data loss). Possibly, one coder was a bit more strict than the other, removing a higher number of automatically detected fixations in the parts where the data was not optimal. This demonstrates that the manual coding can be highly reliable, even in low-quality data sets.

### Comparing hand-coding with the two-step approach

In this section, we demonstrate how the results generated by hand-coding are the same as those obtained applying the two-step approach, where the data are preprocessed using event-detection algorithms before it is hand-coded.

As was mentioned in previous sections, the main purpose of preprocessing the data before they are hand-coded is to speed up the process of detecting fixations: The more fixations the algorithms are able to detect accurately, the less time the coder will expend manually adjusting fixations afterward. It can be argued, however, that having the results from the event detection algorithms as a basis to hand-code fixations can influence the coder’s decisions for accepting or deleting fixations. To demonstrate that this is not the case, we compared results from hand-coding (using GraFIX coding tool, but without preprocessing the data beforehand) with results from the two-step approach.

We used exactly the same data as in the previous section where two groups of infants featuring low- and high-quality data were analyzed. One of the coders recoded all the data using a purely hand-coding approach in order to compare it with results from the previous section coded with the two-step approach.

The interrater reliability between the means and the number of detected fixations was evaluated using the ICC. All the infants were included in the same analysis regardless of their data quality. A strong agreement between the mean fixation durations was found (with an ICC of .993, *p* < .001). Additionally, we also found a strong agreement in the number of detected fixations (with an ICC of .994, *p* < .001).

This analysis demonstrates that the results from a purely hand-coding approach and the two-step approach are the same; thus, both can be considered as close to a “ground-truth” identification of fixations as is possible.

### Comparing the automatic detection with the two-step approach

In this section, we compare GraFIX algorithms for the automatic detection of fixations with the two-step approach where the algorithm’s outcome was also hand-coded.

We used exactly the same data as in the previous sections. In particular, we took exactly the same fixations calculated by one of the raters, which were coded using the two-step approach, and used them to compare with the outcome from the algorithms alone. Since it was demonstrated in the previous section, in terms of results, the only difference between hand-coding and the two-step approach is that the second one is faster. In both cases, the data are manipulated to reach the same criteria; thus, the two-step approach could be considered a method for hand-coding the data. The input values for the automatic detection algorithms were the same as those specified in Table 1.

For the high-quality data group, we found a strong agreement between the automatic and the hand-coding for both mean fixation durations (with an ICC of .973, *p* = .019) and number of fixations (with an ICC of .966, *p* = .008). On the other hand, no significant agreements were found for the low-quality data group for the means (with an ICC of 14.969, *p* = .849), even though there was an agreement in the number of detected fixations (with an ICC of .898, *p* = .073). This can also be seen in the means and standard deviations from Table 2: The values resulting from automatic algorithms and hand-coding in high-precision data look quite similar, whereas it is not the case for low-precision data.

**Table 2:** Automatic versus hand-coding: Fixation duration (FD) means and standard deviations in low- and high-spatial-precision data

	High Spatial Precision	Low Spatial Precision
Automatic algorithms FDs	625.1 ± 847.8 (*N* = 2,410)	552.2 ± 536.3 (*N* = 863)
Two-step approach FDs	627.9 ± 866.2 (*N* = 2,268)	489.9 ± 445.9 (*N* = 858)

**Fig. 6:**
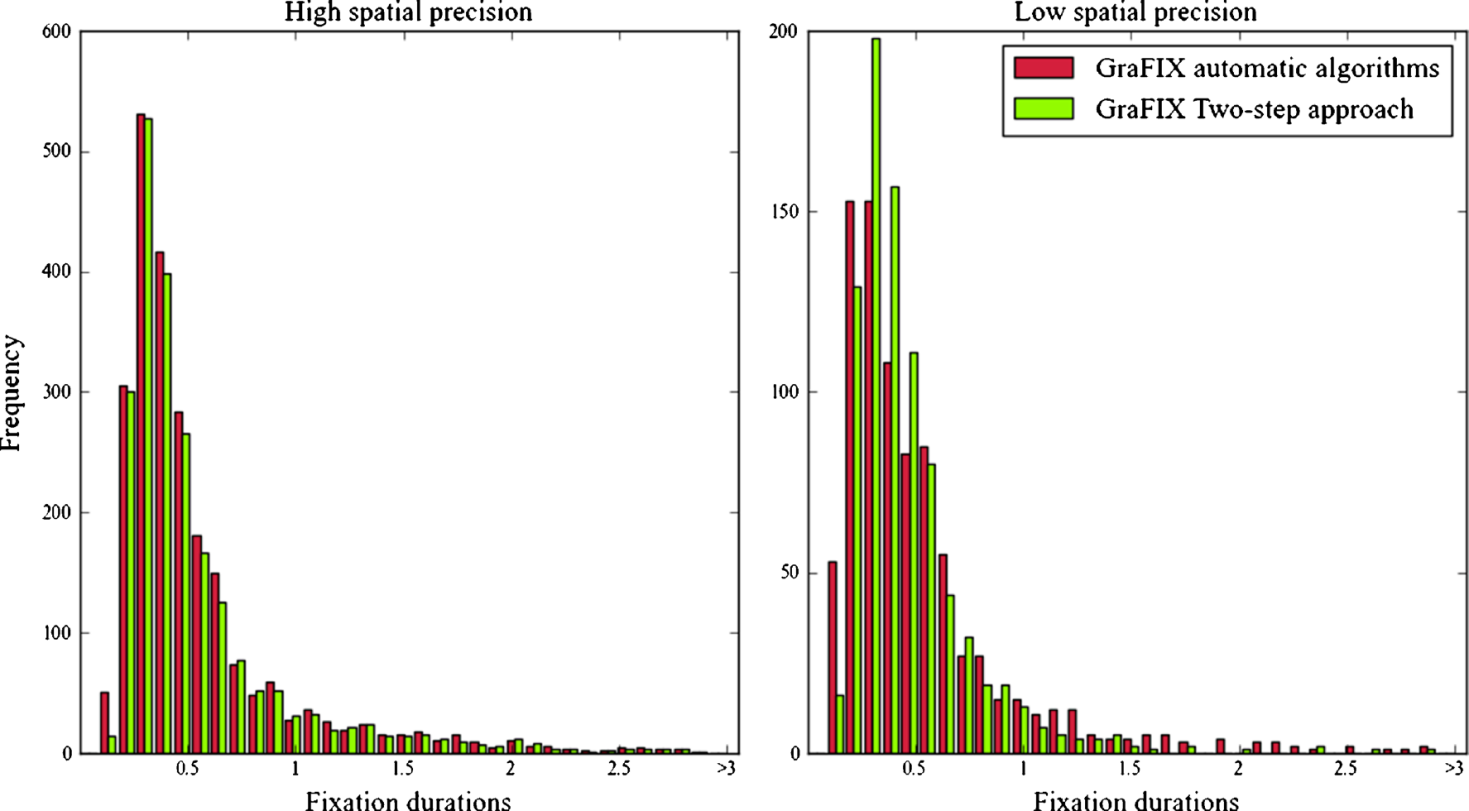
GraFIX automatic algorithms versus two-step approach (automatic detection + hand-coding)

Figure 6 shows how the algorithms are able to accurately detect fixations in high-spatial-precision data (Fig. 6, left), although even then, few manual adjustments are advisable. On the contrary, low-spatial-precision data (Fig. 6, right) need major adjustments, even though these algorithms alone can still capture the trend in the fixation duration distribution.

### Comparing GraFIX with previous approaches

In this section, we compare the detection results from GraFIX (both the automatic algorithms and the hand-coding) with those from previous algorithms. In particular, we tested the fixation-parsing algorithms for low-quality data described in Wass et al. (2013), the adaptive velocity-based algorithms from Nyström and Holmqvist (2010), and the I-VT filter (as implemented in Tobii Studio 3.0.0). The last two algorithms are not designed to deal with particularly low-quality data, such as data recorded from infants. In fact, even though Nyström and Holmqvist (2010) adapt the velocity threshold according to the level of noise, they still maintain that the algorithm is suitable only for data collected from viewers with relatively stable heads while watching static stimuli.^3^ This is obviously not the case for most data coming from infants and other special populations, which is likely to be much noisier than any of the recordings previously tested with these algorithms. However, given that these algorithms are considered a well-established method for event detection, we decided to include them in our comparison.

We selected three infants who presented high-precision data (RMS <0.13° per infant; 5–6 min of data each) and another three infants that presented low-precision data (RMS > 0.25° per infant; 5–6 min of data each) from an experiment that was recorded using a Tobii TX300 eyetracker and Tobii Studio 3.0.0 at a sampling rate of 120 Hz. Once again, it is important to bear in mind that these are data from infants and, thus, still present a high degree of movement, variability in the levels of noise across the experiment, and frequent missing data points, even in the high-precision group.

When possible, we used the same input parameters for the four algorithms (see Table 3). Nevertheless, we still kept two sets of parameters for GraFIX automatic algorithms (for high-precision and low-precision data), since adapting the input values according to the data quality is still one of the main advantages of the present approach.

**Table 3:** Input parameters for the automatic detection algorithms

	GraFIX (High Quality)	GraFIX (Low Quality)	Wass, Smith, & Johnson (2013)	Nyström & Holmqvist (2010)	I-VT Filter
Interpolation latency (ms)	60	60	60	n.a.	60
Velocity threshold (°/sec)	9	20	20	Adaptive	20
Maximum interpolation displacement (°)	0.25	0.25	n.a.	n.a.	n.a.
Degree per pixel (°/pix)	0.0177	0.0177	0.0177	n.a.	n.a.
Maximum distance for merging adjacent fixations (°)	0.24	0.35	n.a.	n.a.	0.35
Maximum time for merging adjacent fixations (ms)	50	50	n.a.	n.a.	50
Maximum RMS per fixation (°)	0.24	0.35	n.a.	n.a.	n.a.
Minimum fixation duration (ms)	99	120	100	100	100

The parameters that we used for the Wass et al. (2013) algorithms and the I-VT filter were, if applicable, the same as for GraFIX algorithms in low-quality data. This is because, when there are participants with various levels of noise, it is a more common practice to use thresholds that rather fit the participants with higher noise. The algorithms from Nyström and Holmqvist (2010) include an adaptive velocity threshold that is recalculated every 10 s (we divided the data for each participant in 10-s chunks). The input parameters that are not reported in Table 3, such as the blink acceleration threshold or the post hoc validation inputs, were set to the values that were recommended in the original articles by Nyström and Holmqvist (2010) and by Wass et al. (2013) respectively.

**Fig. 7:**
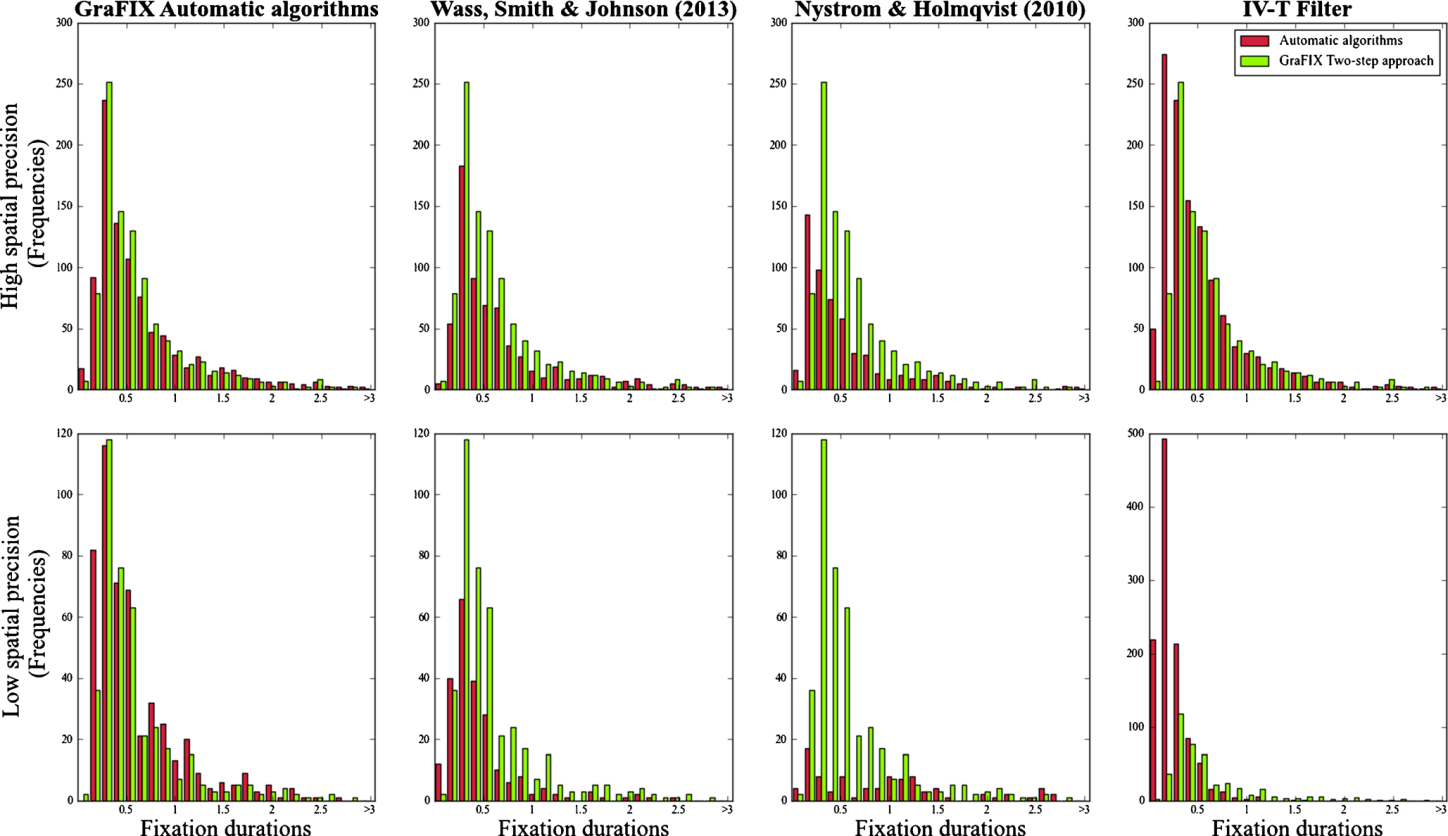
These graphs display the hand-coding (green; GraFIX two-step approach) distribution paired with the distributions for the four different algorithms (red). We assume that the algorithm that is closer to hand-coding is the one able to detect fixations more accurately

Table 4 shows the means and the standard deviations obtained from the different algorithms and hand-coding, and Fig. 7 displays the graphs with all the fixation duration distributions from the four algorithms paired with the hand-coding distribution, which was coded by using the two-step approach. We assume that the algorithm that gets closer to the hand-coding distribution will be the one able to detect fixations more accurately. The differences between algorithms in both high- and low-precision groups are striking. Results for the high-spatial-precision group revealed differences in the means and also in the number of detected fixations. The I-VT filter, in particular, presented an especially high number of detected fixations (*N* = 1199), as compared with hand-coding (*N* = 973), that can be the result of mistakenly flagging very small fixations in segments that were slightly noisier (see Fig. 7, first row, fourth column). For this reason, the mean durations (*M* = 554.0) are lower than the means for the other algorithms or for hand-coding. On the other hand, GraFIX and the Wass et al. (2013) algorithms present means that are a bit above the hand-coding mean (*M* = 674.5). An explanation for this can be related to the selection of the velocity thresholds and the sampling rate. As has previously been mentioned, saccades with very small amplitudes may go undetected by velocity-based algorithms, especially when the data are recorded at low sampling rates (<200). As a consequence, the fixations before and after these saccades will be merged together in a longer fixation. Obviously, at higher velocity thresholds, it is more likely that fixations will be merged together. Since the velocity threshold for Wass et al. (2013) was higher (20 °/sec) than for GraFIX algorithms (9 °/sec), it is also not surprising that the mean for the first algorithms was still higher than for the present algorithms. The algorithms for Nyström and Holmqvist (2010) avoided this problem by adapting the velocity threshold according to the level of noise in the data. Even though they still capture the trend in the distribution, the data quality for the samples that were analyzed (even for the high-precision group) is probably too low to obtain more accurate results from these algorithms. Eyeballing the graphs from Fig. 7 (first row), it is possible to see how the fixation duration distribution produced by GraFIX algorithms had the closest resemblance to the hand-coding distribution in high-precision data.

**Table 4:** Comparing detection algorithms with hand-coding: Fixation durations means and standard deviations in low and high spatial precision data

	High Spatial Precision	Low Spatial Precision
Hand coding	674.5 ± 621.9 (*N* = 973)	657.3 ± 642.0 (*N* = 424)
GraFIX automatic algorithms	719.9 ± 696.4 (*N* = 954)	640.4 ± 589.3 (*N* = 505)
Wass, Smith, & Johnson (2013)	779.3 ± 826.5 (*N* = 676)	491.2 ± 490.2 (*N* = 229)
Nyström & Holmqvist (2010)	571.2 ± 588.7 (*N* = 540)	1,337.9 ± 1,435.1 (*N* =103)
I-VT Filter	554.0 ± 527.4 (*N* = 1,199)	240.7 ± 169.0 (*N* = 1,102)

Results from the low-spatial-precision group revealed even higher differences between the four algorithms. As it can be seen in Table 4 and in Fig. 7 (second row, fourth column), the problem that the I-VT filter presented in the high-precision data was even more obvious here. Likewise, the Nyström and Holmqvist (2010) algorithms did not manage to deal with such a high degree of noise (see Fig. 7, second row, third column). Looking at the graphs and the means, it seems that due to the low precision in the data, the velocity threshold that was calculated may have been too high. It is also interesting to see that even though the length of the recordings was approximately the same for the low- and high-quality groups, the number of detected fixations in the low-precision data was almost half the number of fixations detected in the high-precision data for the GraFIX algorithms, the Wass et al. (2013) algorithms, and hand-coding. The Wass et al. (2013) algorithms exclude a high number of fixations with their post hoc validation criteria, and this is probably why their algorithms still detect many fewer fixations than do the algorithms that we propose. Once again, GraFIX algorithms seem to resemble the hand-coding fixation duration distribution more accurately, even though they would still need manual adjustments (the two-step approach) to be perfect.

Overall, even though all the algorithms are far from perfect, results from GraFIX algorithms were the ones that more closely matched the hand-coding results. We believe that this is not only because of the particularities of our algorithms, but also because we are adapting the input parameters to different levels of noise. We would not recommend, however, the exclusive use of automatic detection algorithms unless the data quality is very high. Evidently, when the algorithms’ outcome is accurate, the time that needs to be invested in correcting artifactual fixations will be considerably lower.

In sum, GraFIX seems to be an effective alternative to previous methods that will improve the quality of our results and the time invested coding eye-tracking data.

## Discussion

In this article, we described a new method and software to parse fixations in low- and high-quality data. Previous fixation detection methods are based on either purely automatic approaches or manual coding of the eye-tracking data. The high variability in data quality across participants and even during the experiment can seriously affect the automatic detection algorithms, and as a consequence, their results can be remarkably unreliable. On the other hand, current hand-coding methods can be extremely time consuming and imprecise. The present method implements a two-step approach for detecting fixations, where the data are first parsed by using a new adaptive velocity-based algorithm especially designed to deal with low-quality data and, second, the algorithm’s outcome is manipulated with a view to fix the errors that the automatic process may have generated.

GraFIX fixation detection algorithms go through a number of steps in order to parse fixations accurately. First, the raw data are smoothed by using a bilateral filtering algorithm (based on previous implementations from Durand & Dorsey, 2002; Frank et al., 2009). This algorithm averages the data for both eyes and eliminates the jitter while preserving saccades. Second, missing data points are interpolated in order to avoid the detection of artifactual fixations in the subsequent steps. We showed how these missing points generate peaks in velocity that can be mistakenly flagged as saccades. Third, a velocity-based algorithm gives us an initial parsing of the data. However, the results from this algorithm may still include artifactual fixations. Finally, GraFIX executes three post hoc validation algorithms aiming to fix and/or remove the artifactual fixations detected in the previous step. In particular, the post hoc algorithms (1) merge adjacent fixations that are close in space and time, (2) remove all the fixations whose RMS is over a given threshold, and (3) delete all the fixations with shorter duration than the minimum fixation (in this order).

GraFIX detection algorithms aim to obtain the most accurate fixation detection for each participant and, thus, reduce the amount of time the researcher has to spend correcting fixations in the subsequent step. Furthermore, the hand-coding graphical tool alone—where the user simply needs to click on the screen to manipulate fixations—is already much faster than previous hand-coding approaches (e.g., coding fixations analyzing videos frame by frame).

We evaluated GraFIX from four different perspectives: (1) We used the ICC in order to evaluate the agreement between two different researchers when coding two groups of infants featuring low- and high-quality data; (2) hand-coding was compared with the two-step approach, demonstrating that both methods generate near identical results; (3) GraFIX automatic algorithms were compared with the two-step approach; and (4) GraFIX automatic algorithms were compared with previous automatic detection methods (the velocity-based algorithms from Wass et al., 2013; the adaptive velocity-based algorithms from Nyström & Holmqvist, 2010; and the I-VT filter). Additionally, GraFIX was tested with data from different eye-tracking systems. Results from these analyses revealed that GraFIX automatic algorithms was the method that more closely matched hand-coding results and that these algorithms alone can be a more reliable technique than other methods, overcoming some of the previous issues detecting fixation in low- and high-quality data. However, we strongly believe that given the nature of our data, any automatic algorithm should be used in combination with a later hand-coding approach.

Many of the current detection algorithms, especially those commercially available, obscure the quality of the data and the fixation detection process. Consequently, evaluating the reliability of results or checking how different parameters affect the fixation detection may become an arduous task. GraFIX allows the adaptation of the input parameters for the automatic algorithms (e.g., velocity threshold, interpolation latency) to fit the data from different participants that present different levels of data quality. As was previously explained, choosing the right velocity threshold highly depends on the quality of the data and the experimental design. Ideally, the user will select low thresholds in order to be able to detect small fixational eye movements. However, the lower the data quality is, the higher the velocity threshold needs to be. If all the participants are at very different levels of data quality, it can be worthwhile to adapt this value on a participant basis. Likewise, the rest of the parameters (such as the post hoc validation parameters) should be adapted according to the participant’s data quality in the interest of obtaining the most accurate results. Additionally, the execution of the algorithms is fast, and it displays all the information that we need to precisely evaluate what the algorithms calculated (interpolation and post hoc validation flags for each point, visualization of the results paired with the velocity and the raw and smoothed data.). This can enormously facilitate the correct selection of the input parameters for the detection algorithm.

Traditionally, all participants are grouped together, and a single velocity threshold that is usually selected to fit low-quality sets is chosen to parse all the data. We have shown how applying high velocity thresholds can be the reason why the algorithms detect artifactual long fixations. We believe that adapting the input parameters on a participant basis can avoid the detection of artifactual fixations and will lead to more accurate and reliable experimental results. However, it is still up to researchers to decide whether they prefer to use the same parameters for all the participants, adapt the parameters on a participant basis, or rather have different input parameters for different groups of participants featuring different levels of data quality (as we did in our validation section). It is also a topic of debate for the field of eye tracking as to how the parameters used to parse each participant’s data should be reported in publications, factored in to statistical analysis, or standardized within populations and across labs.

But to which extent is it acceptable to group together participants with very different data quality? For instance, this can be an important issue in clinical group comparisons where one group may present considerably lower quality data than the other (e.g., ADHD children vs. control groups), when analyzing different age groups (e.g., 3 month olds vs. 14 month olds) or even when comparing “long fixators” with “short fixators.” We know that in low-quality data, there is less probability of finding clean long fixations that can be reliably detected, also, when the data are hand-coded. This can lead to correlations between data quality and fixation durations where low-quality data sets are more likely to present shorter fixations on average. To at least acknowledge these limitations in our studies, it would be advantageous to consistently report data quality measures and detailed descriptions of the detection methods, together with a data quality correlational analysis. Nevertheless, this still does not solve the problem.

Another limitation is related to the way fixations are hand-coded. In the interest of improving the reproducibility of the experimental outcomes, we believe it is very important to include precise guidelines to define how the fixations are being coded. In part, without these guidelines, the intercoder reliability loses its value.

In sum, the proposed method and software prove to be a more reliable and accurate technique for parsing fixations in low- and high-quality data and overcomes many of the issues that previous methods presented. More accurate outcomes and reporting data quality measures and descriptions of the detection methods in scientific papers can considerably improve the viability of research results and, hence, facilitate the replication of previous studies. This can have a big impact not only in research from populations that are particularly difficult to test and that typically present higher degrees of noise (such as infants, people in the autism spectrum, or ADHD patients), but also in participants that simply do not reach certain data quality standards. In fact, nowadays we are experiencing an increase in the number of new low-cost eye-tracking systems that inherently suffer from data quality issues even with compliant participants. Additionally, GraFIX could also be adapted to code data from head-mounted eye-tracking systems by including the head position and the eye and scene images.

## Software download

Please download GraFIX from: http://sourceforge.net/projects/grafixfixationscoder/

## Appendix 1

### Application overview

The main window consists of a number of boxes where the eye-tracking data and the application’s output is visualized, in addition to all the necessary buttons used to manipulate fixations (see Fig. 2). A brief explanation of these different components is included below.

1. Menu bar. The menu bar allows access to different dialogs such as *Project configuration*, *Visualizations*, or *Automatic Detection of Fixations* dialogs.
2. Raw data. The horizontal axis represents time, while the vertical axis displays the position of subsequent gaze data-points (Each data-point consists of the *x*- and *y*-coordinates for the right and/or the left eye.). For binocular systems, the right- and left-eye coordinates are displayed (*x*-right = red; *x*-left = orange; *y*-right = dark blue; *y*-left = light blue). In case the system is monocular, the data from the second eye will simply not be displayed.
3. Smoothed data. As for the raw data, the horizontal axis represents time, while the vertical axis displays the position of subsequent smoothed gaze data-points. Once the data is smoothed, the resulting *x*- and *y*-coordinates are displayed.
4. Pupil dilation. In cases where the eye-tracking system provides pupil dilation data, it is displayed in this box. Further, the average pupil dilation is calculated for each fixation.
5. Velocity. The horizontal axis represents time, while the vertical axis displays the velocity for subsequent gaze data-points.
6. Missing data from left and right eyes. If there is a data-point missing for either of the eyes, it is displayed in this box.
7. Automatically detected fixations. The orange boxes represent the fixations that are automatically detected by the automatic-detection algorithms.
8. Flags from the automatic-detection algorithm. During the automatic detection, the data are interpolated, and the post hoc validation criteria are applied. The flags indicate which data-points were affected by different algorithms.
9. The cursor’s current location. A horizontal line is displayed at the cursor’s location, facilitating the accurate coding of fixations’ onsets and offsets. Additionally, the *From* label indicates the line in the raw file where the cursor is located.
10. Manually coded fixations. The green boxes represent the fixations that can be manipulated manually. Each fixation has a fixation number that matches the numbers from the first column at the fixations list.
11. Fixations list. This is a list of the fixations that are being manually coded (green boxes). The first column shows the fixation number, the second displays the line in the raw file where the fixation starts, the third displays the line in the raw file where the fixation ends, and finally, the fourth column reveals the fixation duration in seconds.
12. Select the action button. In order to perform an action, the corresponding option has to be selected. Once it is selected, one will need to click and drag the cursor in order to create, delete, or merge fixations. Moreover, it is possible to target smooth-pursuit fixations by selecting this option and dragging the cursor on top of the fixations that need to be coded. In order to add more precision to the task, it is possible to enter in the textboxes the start and the end of the fixation that needs to be manipulated and press *Execute*. This will execute the action currently selected using the specified start and end points.
13. Nonactive segment. The portion of the screen in gray corresponds to the tasks in the experiment that do not need to be coded.
14. Active segment. The portion of the screen in white corresponds to the tasks in the experiment that need to be coded.

### Input files

The *raw input file* is a csv file separated by commas with the following columns: (1) time in microseconds; (2) zeros; (3) relative gaze-point *x* left eye; (4) relative gaze-point *y* left eye; (5) relative gaze-point *x* right eye; (6) relative gaze-point *y* right eye; (7) pupil diameter left eye (optional); and (8) pupil diameter right eye (optional).

If the eye-tracking system is monocular, all the columns corresponding to the second eye should be substituted with −*1 s*.

The *segments input file* indicates which parts of the experiment need to be coded. If the whole data file is the subject of interest, this file does not need to be included.

The segments input file is a csv file separated by commas with the following columns: (1) segment id, which is an unique number for each row; (2) row number in the raw file where the segment starts; (3) row number in the raw file where the segment ends.

### Output files

#### File smooth_[subject number].csv

This file is created when the data is smoothed. Each row corresponds to a data point from the raw file; thus, both files have the same length. This file is a csv file separated by commas with the following columns: (1) time in microseconds; (2) zeros; (3) smoothed *x*-coordinate; (4) smoothed *y*-coordinate; (5) velocity; (6) Is saccade flag: 0/1; (7) Is interpolated flag: 0/1; (8) post hoc merge adjacent fixations flag: 0/1; (9) post hoc RMS flag: 0/1; (10) post hoc minimum fixation flag: 0/1.

#### File fix_auto_[subject number].csv

This is created when fixations are automatically estimated. Each row contains the information for one fixation. This file is a csv file separated by commas with the following columns: (1) row from the raw file where the fixation starts; (2) row from the raw file where the fixation ends; (3) duration in seconds; (4) Average *x*-coordinate; (5) Average *y*-coordinate; (6) RMS; (7) Is smooth pursuit flag: 0/1; (8) Average pupil dilation.

#### File fix_all_[subject number].csv

This file is generated when the first fixation is created, and it is updated every time a fixation is manipulated. It is a csv file separated by commas with the following columns: (1) row from the raw file where the fixation starts; (2) row from the raw file where the fixation ends; (3) duration in seconds; (4) Average *x*-coordinate; (5) Average *y*-coordinate; (6) RMS; (7) Is smooth pursuit flag: 0/1; (8) Average pupil dilation.

## Appendix 2

### Pseudo-code of the proposed interpolation algorithm

1. Calculate the velocities for each point
2. Iteratively find velocity peaks and flag those over the velocity threshold as saccades
3. Find next gap
  3.1. If the gap is longer than the interpolation latency:
    3.1.1. Calculate the Euclidean distances from a central point for the fixations preceding and following the gap.
    3.1.2. If the difference between both Euclidean distances is lower than the maximum displacement threshold:
      3.1.2.1. Interpolate.

Pseudo-code of the proposed velocity algorithm and the post hoc validation

1. Calculate the velocities for each point
2. Iteratively find velocity peaks and flag those over the velocity threshold as saccades
3. The data points in between two saccades are grouped and flagged as fixations
4. Post hoc validation: Merge adjacent fixations with similar location
  4.1. If this post hoc validation criterion is selected
    4.1.1. Find next two adjacent fixations
    4.1.2. If the gap between the two fixations is shorter then 50 ms
      4.1.2.1. Calculate the distance in degrees between the locations for the two fixations
      4.1.2.2. If the distance is shorter than the maximum displacement threshold
        4.1.2.2.1. Merge both fixations
5. Post hoc validation: The RMS of intersample distances per fixations does not exceed a threshold
  5.1. If this post hoc validation criterion is selected
    5.1.1. Find all fixations with an RMS over the threshold
    5.1.2. Delete them
6. Post hoc validation: Minimum fixation duration
  6.1. If this post hoc validation criterion is selected
    6.1.1. Find all fixations with a duration over the threshold
    6.1.2. Delete them

## Footnotes

1 See Psychophysics Toolbox documentation: http://psychtoolbox.org/.

2 See T2T documentation: http://psy.ck.sissa.it/t2t/.

3 See the README attached to the code the authors provide.
